# Effectiveness and Safety of Niraparib as Neoadjuvant Therapy in Advanced Ovarian Cancer With Homologous Recombination Deficiency (NANT): Study Protocol for a Prospective, Multicenter, Exploratory, Phase 2, Single-Arm Study

**DOI:** 10.3389/fonc.2022.852772

**Published:** 2022-03-23

**Authors:** Dongchen Zhou, Jiahao Liu, Ronghua Liu, Huayi Li, Yi Huang, Ding Ma, Li Hong, Qinglei Gao

**Affiliations:** ^1^ Department of Gynecological Oncology, Tongji Hospital, Tongji Medical College, Huazhong University of Science and Technology, Wuhan, China; ^2^ National Clinical Research Center for Obstetrics and Gynecology, Cancer Biology Research Center (Key Laboratory of the Ministry of Education), Tongji Hospital, Tongji Medical College, Huazhong University of Science and Technology, Wuhan, China; ^3^ Department of Gynecological Oncology, Hubei Cancer Hospital, Tongji Medical College, Huazhong University of Science and Technology, Wuhan, China; ^4^ Department of Gynecology and Obstetrics, Renmin Hospital of Wuhan University, Wuhan, China

**Keywords:** ovarian cancer, HRD, neoadjuvant therapy, niraparib, phase II study, single-arm

## Abstract

**Background:**

Ovarian cancer (OC) is a heterogeneous gynecological malignancy with a poor prognosis as the majority of patients are diagnosed at an advanced stage. Neoadjuvant chemotherapy (NACT) followed by interval debulking surgery (IDS) is recommended for patients who cannot achieve optimal cytoreduction or cannot endure primary debulking surgery (PDS). As there is an increased risk of chemoresistance for platinum-based NACT, it is important to investigate an alternative option. A Poly (ADP-ribose) polymerase inhibitor (PARPi), niraparib, has shown high anti-tumor activity, especially in homologous recombination deficiency (HRD) positive patients with OC. Thus, niraparib as a neoadjuvant treatment agent may help improve surgery accessibility and create survival benefits.

**Methods:**

This multicenter, prospective, single-arm, open-label, phase II study plans to recruit 53 patients (aged 18-75 years) with newly diagnosed HRD positive, unresectable (Fagotti score ≥ 8 or upper abdominal computed tomography [CT] score ≥ 3) International Federation of Gynecology and Obstetrics (FIGO) stage III-IV OC. The HRD status was detected by next-generation sequencing and HRD positive patients will be counseled for study participation. Enrolled patients will receive niraparib capsules QD (200mg or 300mg per day) for two cycles (4 weeks/cycle). After neoadjuvant niraparib treatment, patients exhibiting complete response (CR), partial response (PR), or stable disease (SD) will undergo tumor reduction surgery and subsequent standard carboplatin/paclitaxel-based chemotherapy. The primary objectives include the objective response rate (ORR) and R0 resection rate. The rate of treatment interruption/termination and progression-free survival (PFS) will be secondary objectives. The study uses Simon’s optimal two-stage design (24 and 21 patients for the first and second stage respectively). The data manager will record all adverse events (AEs).

**Discussion:**

This is the first prospective study to evaluate the effectiveness and safety of niraparib in neoadjuvant treatment for advanced OC. The result of this study will provide a solid base for further expanding the clinical applications of the PAPRi and exploring more therapeutic possibilities for patients with HRD positive advanced OC. **Clinical Trial Registration**: https://clinicaltrials.gov/, identifier NCT04507841.

## Introduction

Cancer statistics in China indicated that there is an increasing incidence and mortality due to cancer with about 55,342 (17.6%) new ovarian cancer (OC) cases and 37,519 (18.1%) deaths in 2020 (1). The world OC coalition 2020 reported China with the largest number of women with OC in terms of incidence and 5-year prevalence (2). The majority of OCs are at an advanced stage corresponding to stages II b to IV of the International Federation of Gynecology and Obstetrics (FIGO) classification, resulting in poor prognosis (3, 4). Advanced stage presentation has a 5-year relative survival rate of 29% (5). The size of the residual lesion after surgery is an important prognostic factor for survival, so the 5-year survival rate is even worse for those who cannot receive optimal resection.

Standard therapy as per NCCN guidelines for patients with OC (2021) includes surgical debulking or cytoreductive surgery (residual disease <1 cm [R1] and removal of macroscopic disease [R0]) followed by platinum-based chemotherapy (6, 7). If optimal resection (R0 and R1) cannot be achieved, platinum-based neoadjuvant chemotherapy (NACT) with interval debulking surgery (IDS) should be considered (6). Sub-optimal debulking and platinum resistance predominantly leads to treatment failure and high mortality. Hence, complete R0 resection and platinum sensitivity are important for prolonging survival (8). NACT increased the chances of complete resection thereby resulting in improved progression-free survival (PFS) and overall survival (OS) theoretically (9). However, according to previous reports, survival benefits brought by increased R0 resection rate may be diminished by NACT-induced platinum resistance. Evidence showed that NACT may enhance cancer cell stemness, which may lead to chemoresistance, and patients who underwent NACT may experience more platinum resistance and shorter platinum-free interval for recurrence (10, 11). As the platinum-containing regimen in NACT may induce resistance at a later stage, therefore it is important to use an alternative non-platinum-based NACT to avoid platinum chemoresistance and to produce complete R0 resection opportunity in patients with advanced OC.

Evidenced by multiple clinical trials, poly (ADP-ribose) polymerase (PARP) inhibitors (including olaparib, niraparib, veliparib, etc.) have revolutionized the treatment paradigm of OC by eliminating HRD positive or *BRCA 1/2* mutated tumors (12–15). The anti-cancer activities of PARP inhibitors have been confirmed step by step from the late-line to the front-line maintenance therapy (16). Among these drugs, niraparib, an efficient FDA (Food and Drug Administration)-approved *PARP 1/2* inhibitor, showed competence as monotherapy for the late-line treatment of OC, with an overall response rate of 28% in homologous recombination deficiency (HRD) positive patients (95% CI 15.6-42.6; one side P=0.00053) (17). Based on the QUADRA trial, the FDA approved niraparib to treat HRD positive ovarian cancer patients with platinum-sensitive relapse after ≥3 line chemotherapy. Thus, niraparib might serve as an alternative agent for platinum-based NACT in patients with HRD positive OC. Besides, chemotherapy-naïve OC was more sensitive to platinum, as compared to recurrent tumors, implying the potential for a better anti-cancer efficiency of niraparib if served as neoadjuvant therapy.

Therefore, it can be hypothesized that applying neoadjuvant niraparib could reduce platinum resistance and ensure maximum benefit from cytoreductive surgery and postoperative chemotherapy in ovarian cancer patients with HRD positive, unresectable or intolerable to surgery, and ultimately lead to improved prognosis. And the present study is conducted aiming to assess the efficiency and safety of niraparib in newly diagnosed advanced OC.

## Methods

### Study Design

This multicenter, prospective, interventional, single-arm, open-label, phase II study plans to recruit 53 women from 10 centers from China. This study will be conducted in accordance with the protocol, the current version of the Declaration of Helsinki, Good Clinical Practices (GCP) guidelines and any local regulations. The study has been registered at ClinicalTrials.gov (NCT04507841) was approved by the China-South East and Middle Gynecological Oncology Group (CSEM GOG-017). This trial was also approved by the Research Ethics Commission of Tongji Medical College of Huazhong University of Science and Technology (2020-S122). Informed consents will be obtained from patients both before screening and before receiving niraparib treatment.

**Figure 1:**
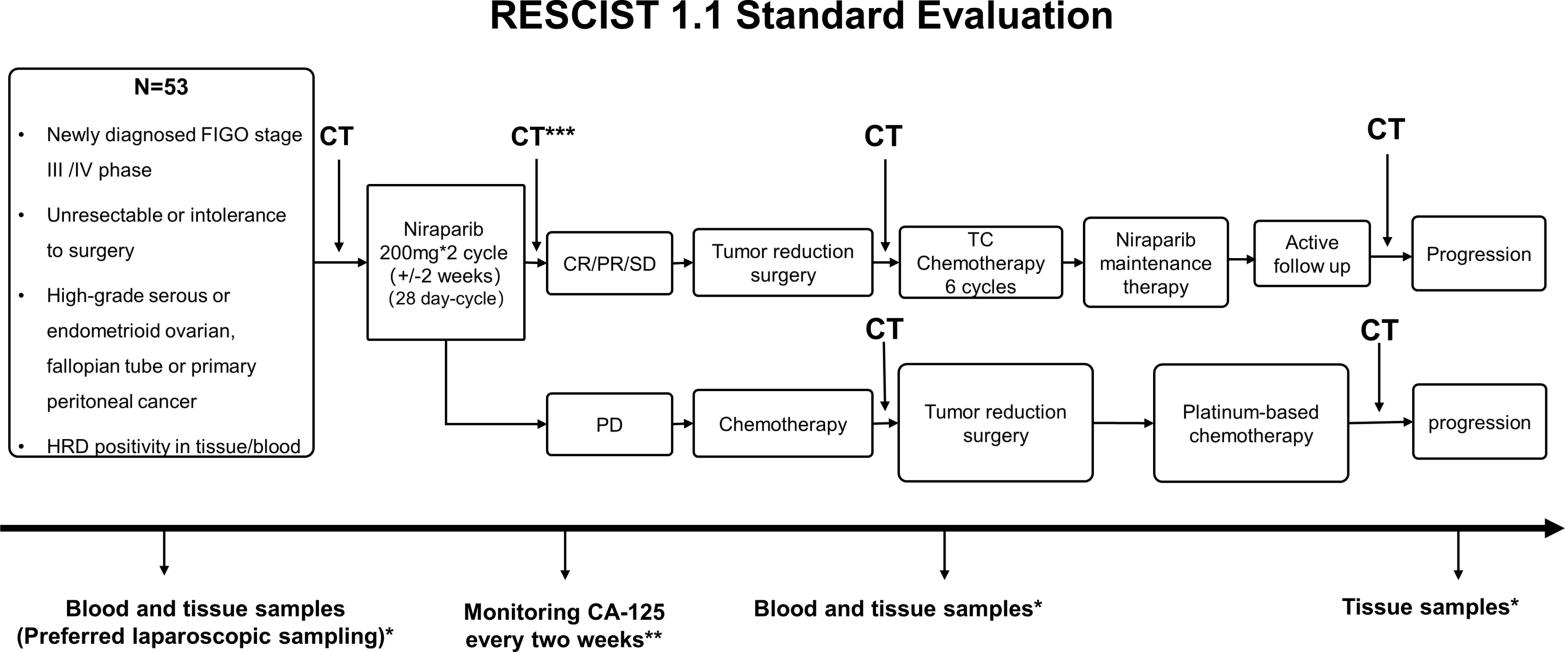
Study Design. *Blood and tissue samples were collected at various stages i.e., before and during treatment, disease progression/recurrence, during surgery and post-operative chemotherapy and follow-up. **CA-125 was determined as per Gynecologic Cancer Intergroup Consensus (GCIC) guidelines. ***Abdominal CT or laparoscopy is recommended to classify objective remission status. CA-125, cancer antigen-125; CR, complete response; CT, computed tomography; FIGO, International Federation of Gynecology and Obstetrics; HRD, homologous recombination deficiency; NACT, neoadjuvant chemotherapy; N, number; PD, progressive disease; PR, partial response; RECIST, Response Evaluation Criteria in Solid Tumors; SD, stable disease; TC, Paclitaxel and carboplatin regimen.

The study design is provided in **Figure 1**. Treatment-naïve patients (aged 18-75 years) with newly diagnosed, HRD positive, low likelihood of optimal cytoreduction by computed tomography evaluation ≥3 or Fogotti score ≥8, FIGO stage III-IV OC will be included. The detailed inclusion and exclusion criteria are provided in **Table 1**. Informed consent will be obtained before HRD testing and receiving niraparib treatment, and the choice of treatment will depend on patients’ preferences. All enrolled patients will receive a minimum of 200mg or 300mg QD of niraparib as 100 mg capsules for 2 cycles each lasting for 28 days. The initial dose will be adjusted according to the baseline body weight and platelet count (≥77 kg and 150000/UL, 300 mg dose is recommended; otherwise 200 mg dose). After receiving niraparib for 2 cycles, abdominal computed tomography (CT) scan will be performed to classify objective remission status according to Response Evaluation Criteria in Solid Tumors (RECIST version 1.1). If patients achieve complete response (CR), partial response (PR) or stable disease (SD), they will receive open laparotomy IDS. Post-surgery, routine platinum-based chemotherapy will be given for 6 cycles, following which, niraparib will be given as maintenance therapy within 12 weeks up to 3 years or till disease progression or patient withdrawal from the study since they are high risk of recurrence and demonstrated efficacy in neoadjuvant therapy. However, patients with progression disease (PD) will receive NACT and follow-up therapy as recommended by the NCCN guidelines. In the case of grade 3-4 adverse events (AEs), the treatment should be suspended, and the AEs should be actively treated until returning to grade 1-2. The dose may be reduced in the next cycle of treatment depending on the decision of the investigators. If the toxicity does return to grade 1-2 or below within 28 days, no further reduction below 100mg/day will be allowed.

**Table 1:** Key inclusion and exclusion criteria.

Inclusion Criteria	Exclusion Criteria
Women patients aged 18-75 years	Personnel involved in the planning or implementation of the research
High-grade serous or endometrioid ovarian/peritoneal/fallopian tube cancer, FIGO stage III-IV confirmed by open or laparoscopic surgery or coarse needle aspiration biopsy	Patients participating in other clinical drug experiments or administering other research drugs or neoadjuvant therapies (chemo/radio/immuno therapies, TCM) at the same time as the study
HRD positive confirmed by tissue/blood samples	Allergy to niraparib or with similar chemical/biologic analogs
Consent for providing tissue/blood samples not only during the course of treatment but also for expanded gene/tumor markers related research studies	Dysphagia or any other GIT condition interfering with the ADME of the drug
At least one lesion measurable by CT/MRI	Previously received any treatment or PARPIs for ovarian cancer
Failure to achieve R0 tumor reduction (Fagotti score ≥8, upper abdominal CT score ≥3) or surgery intolerance (age ≥80 years, BMI ≥40, chronic diseases, malnutrition or hypoproteinemia, moderate to massive ascites, newly diagnosed venous thromboembolism, ECOG >2)	Simultaneous treatment of symptomatic or uncontrolled brain metastases requiring surgery, radiation and/or corticosteroids, or clinical manifestations of spinal cord compression
Expected survival time: >12 weeks	Non-recovery from a major surgery performed within 3 weeks before the start of the study
ECOG score: 0-2	Previous or current diagnosis of MDS/AML or other primary malignancies, except for carcinoma *in situ* of the skin’s basal and squamous, breast ductal, or cervix
Good organ function: **─** Bone marrow (neutrophil count ≥1500/μL, platelet ≥100000/μL, Hb ≥10g/dL) ** ─** Liver (Total bilirubin ≤1.5 times, direct bilirubin ≤1 time, AST and ALT ≤2.5 times, if liver metastasis exists ≤5 time of the upper limit of normal value) **─** Renal (Serum creatinine ≤1.5 times the upper limit of normal value, or creatinine clearance rate ≥60 ml/min)	Disease or conditions exposing patients to high-risk toxicity, including HIV, hepatitis B and C; severe cardiovascular disease, intractable ventricular arrhythmias myocardial infarction in the last three months; uncontrolled epileptic grand mal seizure, unstable spinal cord compression or superior vena cava syndrome; psychiatric disorders affecting patients’ informed consent; hypertension beyond drug control or unsuitable for participation in the study identified by researchers	
Fertile women must have negative pregnancy tests, adequate contraception (except hormonal) within one week before enrolment and should be non-lactating; women without reproductive potential are also eligible (menopause/surgical sterilization)	Medical history or existing clinical evidence likely to interfere with study results or patients’ compliance
Sound understanding and ability to comply with the procedures involved in study protocol such as treatment schedule, laboratory testing, imaging testing, follow-up and willingness to complete questionnaire survey of quality of life	Platelet or red blood cell transfusion within 3 days before the start of treatment of the study drug
Previous chemotherapy toxicity should be ≤ CTCAE 1 or baseline level, except for sensory neuropathy or alopecia with stable symptoms ≤ CTCAE grade 2	Clinical unresolved toxicity ≥ grade 2, except neuralgia, lymphopenia, and depigmentation of skin

ADME, absorption distribution metabolism excretion; ALT, alanine aminotransferase; AML, acute myeloid leukemia; AST, aspartate aminotransferase; BMI, body mass index; CT, computed tomography; CTCAE, Common Terminology Criteria for Adverse Events; ECOG, Eastern Cooperative Oncology Group; FIGO, The International Federation of Gynecology and Obstetrics; GIT, gastrointestinal tract; Hb, hemoglobin; HIV, human immunodeficiency virus; HRD, homologous recombination deficiency; MDS, myelodysplastic syndrome; MRI, magnetic resonance imaging; PARPis, Poly (ADP-ribose) polymerase inhibitors; TCM, traditional Chinese medicine.

### Study Objectives and Endpoints

The primary objective is to evaluate objective response rate (ORR) and R0 resection rate after niraparib neoadjuvant treatment. ORR is defined by the rate of patients achieving CR or PR. The secondary endpoints will be to evaluate the number of patients with treatment-related AEs or serious AEs as assessed by Common Terminology Criteria for Adverse Events (CTCAE version 5.0). Furthermore, the rate of treatment interruption and termination caused by patients’ intolerance of side effects, incidence of AEs at all levels during the course of the treatment, surgery and chemotherapy following treatment will also be determined. The secondary efficacy endpoints will be to determine disease control rate (DCR); pathologic complete response rate measured by Miller-Panye system; PFS; cancer antigen 125 (CA125) progression rate as per Gynecologic Cancer Intergroup Consensus (GCIC) guidelines; OS and long-term benefits including chemotherapy-free interval (CFI) and time to first subsequent therapy (TFST). The definitions of primary secondary endpoints are provided in **Table 2**.

**Table 2:** Primary and secondary endpoints.

Primary endpoints	Definition
R0 resection rate*	The percentage of initially unresectable patients who successfully achieve R0 resection.
ORR	The percentage of patients who experienced complete or partial remission after niraparib neoadjuvant niraparib treatment with evaluated by the RECIST1.1 criteria.
**Secondary endpoints**	
PFS	Time from receiving Niraparib to tumor progression or cancer-related death as assessed by RECIST version 1.1.
DCR	The proportion of patients achieving CR, PR and SD.
OS	Time between receiving Niraparib and death by any cause.
pCR	Complete disappearance of the tumor cells in surgical specimens after niraparib neoadjuvant therapy.

*R0 resection indicates microscopically margin-negative resection, in which no gross/microscopic tumor remains in the primary tumor bed.

CR, complete response; DCR, disease control rate; OS, overall survival; PFS, progression-free survival; PR, partial response; RECIST, Response evaluation criteria in solid tumors; SD, stable disease; pCR, complete pathologic response.

For assessing quality of life (QOL), validated patient report outcome (PRO) tools will be used namely, Functional Assessment of Cancer Therapy-Ovarian (FACT-O), Hospital Anxiety and Depression Scale (HADS), Insomnia Severity Index (ISI), International Physical Activity Questionnaire (IPAQ), EuroQol-visual analog scales (EQ-VAS).

The exploratory objectives will include changes in tumor biomarkers such as CA-125 during treatment, exploring biomarkers related to efficacy and patient prognosis and changes in gene mutation before and after treatment from tumor tissue and blood.

### Visiting Plan

**Figure 2:**
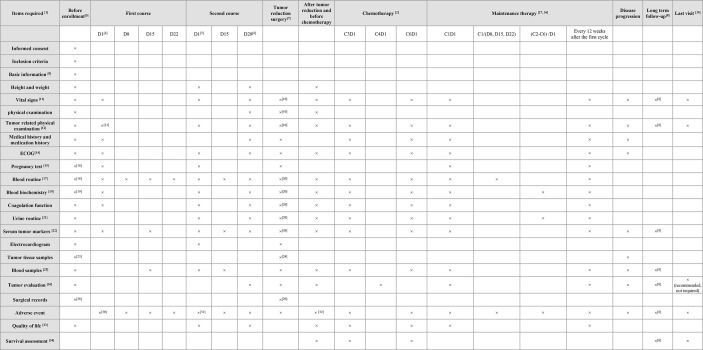
Follow-up plan. ^1^In addition to the prescribed visit schedule, local researchers may conduct more frequent inspections as per patients’ requirements, which may include blood and urine routine examination, blood biochemistry, ECG, CT, and serum tumor biomarker (within one week); ^2^Refers to platinum-based chemotherapy post-surgery and included information collected from the patient’s medical record; ^3^It should not be completed >4 weeks before enrolment, excluding exceptional cases; ^4^Required to be completed within 72 hours of the start of the cycle 1 ( ± 1D); ^5^Required to be completed within 72 hours of the start of the cycle 2 ( ± 1D); ^6^Required to be completed within 72 hours from the beginning of cycle 2 treatment D28 (± 1D); ^7^Required to be completed within 3 days pre-operation; ^8^Once every 12 weeks after maintenance treatment and once every 24 weeks after two years; ^9^Includes name, age, gender, place of origin, contact details and date of admission; ^10^Time interval from last inspection should be >7 days, otherwise the inspection shall be canceled; ^11^Includes heart rate, blood pressure, pulse and respiration; ^12^Records the tumor size at least; ^13^Recommended, non-mandatory; ^14^The United States Eastern Cooperative Oncology Group (ECOG) physical status scores; ^15^Blood or urine β-HCG test; ^16^Completed within two weeks before enrollment; ^17^Includes neutrophil/platelet count, and Hb level; ^18^Atleast 7 days before enrollment; ^19^Includes measurement of serum creatinine and electrolytes, total bilirubin, ALT/AST; ^20^Time interval from the last inspection should not be <1 week, otherwise the inspection shall be canceled; ^21^Includes measurement of creatinine, urea nitrogen, and erythrocytes; ^22^Includes testing of CA125, CA199, CEA, and HE4 related markers; ^23^Biopsy done by laparoscopy, laparotomy, or coarse-needle aspiration; ^24^Primary and metastatic tumor tissues obtained by laparotomy/laparoscopic tumor reduction surgery; ^25^Samples in 2ml EDTA anticoagulant tube were used for HRD detection before enrollment and after two courses of treatment. Samples of 10ml Streck tube were collected before enrollment, D15 and D28 of the first course of chemotherapy, D15 of the second course of chemotherapy, and before tumor reduction surgery and subsequent therapy; ^26^Abdominal CT or MRI is recommended for evaluation instead of ultrasound alone; ^27^If blood routine tests are abnormal during maintenance treatment, then it should be carried out every 3 days and closely monitored until it becomes normal; ^28^The operation record of the biopsy should be sent to the research center for record within 7 days; ^29^Surgical records should be completed within 24 hours after the surgery and sent to the research center within 7 days for reference; ^30^All adverse events to be documented from first day of receiving niraparib to post 30 days of treatment termination; ^31^All adverse events to be documented from first day of receiving niraparib to post 30 days of treatment termination; ^32^Postoperative adverse events (D1 to D28); ^33^Includes FACT-O, HADS, ISI, IPAQ, and EQ-VAS; ^34^Recurrence and time of recurrence, death and time of death, whether to continue follow-up and last follow-up time are recorded; ^35^The last follow-up before withdrawal; ^36^After the completion of first-line chemotherapy, patients will receive maintenance treatment with niraparib within 12 weeks. ALT/AST, alanine aminotransferase/aspartate aminotransferase; CA, cancer antigen; CEA, carcinoembryonic antigen; CT, computed tomography; D, day; ECOG, Eastern Cooperative Oncology Group; ECG, electrocardiogram; EDTA, ethylenediaminetetraacetic acid; EQ-VAS, EuroQol-visual analog scales; FACT-O, Functional Assessment of Cancer Therapy-Ovarian; HADS, Hospital Anxiety and Depression Scale; Hb, hemoglobin; β-HCG, β-human chorionic gonadotropin; HE4, human epididymis protein 4; HRD, homologous recombination deficiency; IPAQ, International Physical Activity Questionnaire; ISI, Insomnia Severity Index; MRI, magnetic resonance imaging.

HRD positive status will be screened by the next generation sequencing technology on peripheral blood and tumor tissue samples by using three classic indicators of genomic instability (LOH, loss of heterozygosity; TAI, telomeric allelic imbalance and LST, large scale state transitions). It takes 7 natural days from sampling to issuing the report, the same time as routine postoperative examination. Baseline data will be recorded at the screening visit, and it must be completed within 28 days before enrolment. Pre-assessment on the basis of parameters specified in **Figure 2** will be performed within 3 days before tumor reduction surgery.

Patients will be followed up every three months during the first two years of the treatment, every six months for 2-5 years, and every year thereafter. Information on disease progression, safety and complications will be collected during follow-up and further recorded in the case report form (CRF). The treatment and follow-up plan has been presented in **Figure 2**. Survival follow-up will be conducted every 90 days (± 7 days) after drug withdrawal. Moreover, information of patients receiving new chemotherapy for the first time after the end of this study will be collected. Treatment can be terminated at any point during the study due to any of the following reasons: serious/life-threatening or intolerable treatment-related AEs, risk to patients, protocol violation, withdrawal of consent, pregnancy, and disease progression. The end of treatment (EOT) and follow-up visit should be completed if the study is discontinued or in case of patient’s withdrawal from the study. The following categories of concomitant medications are prohibited during the study period: granulocyte colony-stimulating factor (G-CSF), CYP1A2 sensitive substrates, anticoagulants and antiplatelet drugs, systemic glucocorticoids, other PARPis, radiation therapy, vaccines and hormonal contraceptives.

### Effectiveness Evaluation

Computed tomography (CT) of the abdominal and pelvic cavity and other tumor areas with clinical indications will be performed at baseline and each follow-up (**Figure 2**). Tumor radiological imaging will use RECIST v1.1 criteria to classify objective remission status. Target lesions will be classified into CR, PR, SD, PD and non-evaluable (NE). Non-target lesions will be evaluated on the basis of CR, non-CR/non-disease progression (NN), PD, and NE.

Tumor response will be classified as PD for target lesions if there is ≥ 20% increase in the sum of the longest diameter of target lesions in comparison to the smallest sum longest diameter recorded in addition to an absolute increase of 5 mm whereas in case of non-target lesions, appearance of one or more new lesions or unequivocal progression of existing lesions will be considered as PD. New lesions refer to the appearance of new malignant lesions indicative of PD (18). In the case of PD, treatment should be terminated, and the follow-up should be continued. Target lesions of all patients will be measured by the same imaging technique and recorded in the CRF. Patients receiving at least two treatment cycles and one disease assessment or patients with early progression will be considered evaluable. Full analysis set (FAS) used for effectiveness evaluation will include all patients with primary gynecological malignancies (sub-grouped by tumor type), meeting the inclusion-exclusion criteria, with complete medical records.

### Data and Sample Collection, Management, and Monitoring

**Figure 3:**
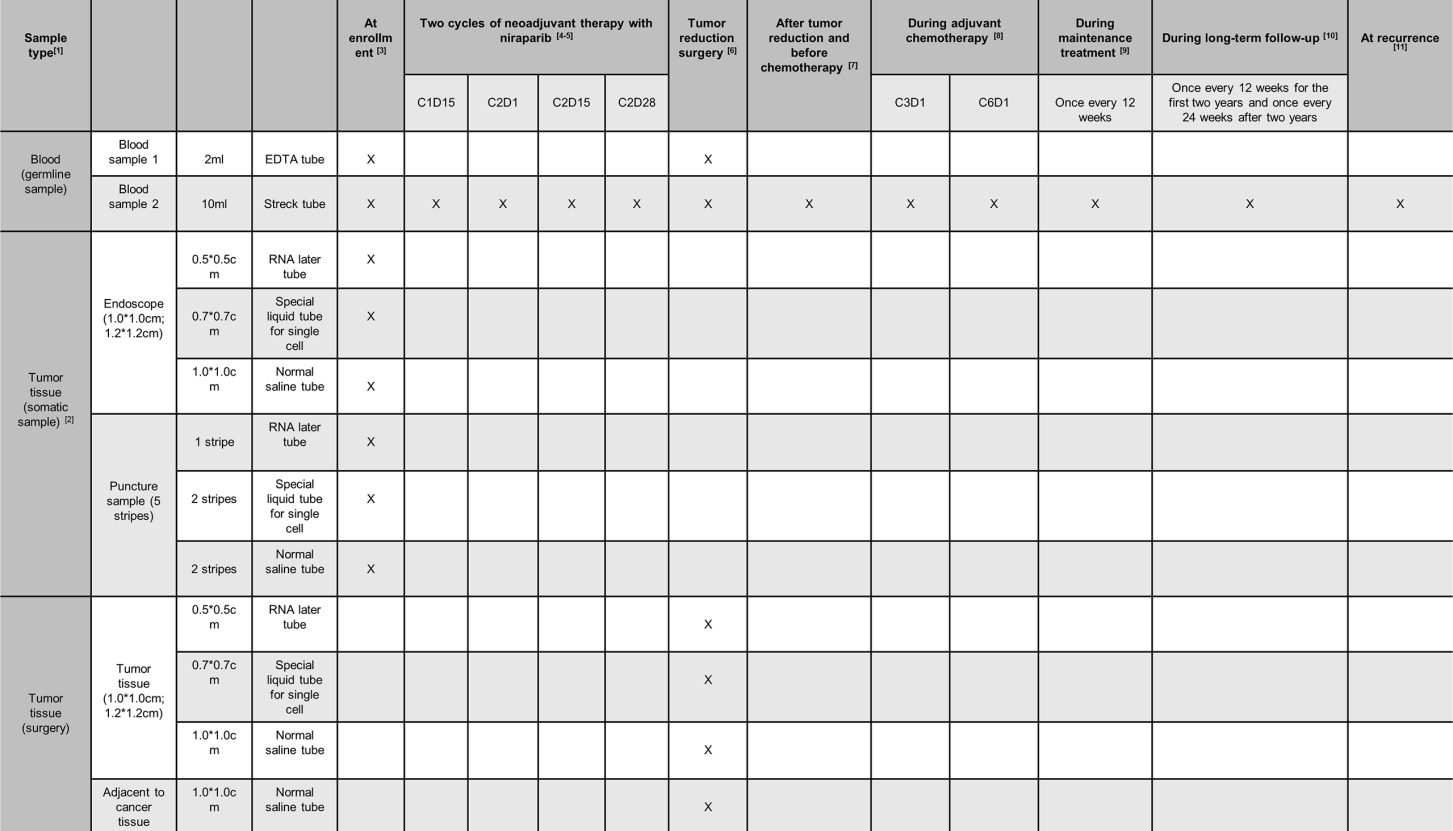
Sample collection plan. ^1^In addition to pathological sections, one copy of frozen, paraffin-embedded tissue for gene detection, and RNA preservation samples will be sent for sample retention. Also, samples in 2ml EDTA anticoagulant tube and in 10 ml streck tube should be sent to Tongji laboratory immediately for ctDNA sequencing; ^2^Tissue sample specimens should first meet the requirements of normal histopathological examination, and the remaining samples should be subjected to gene detection and tissue preservation according to the sample collection process; ^3^Tissue samples should be obtained at the time of biopsy. Sample in 2ml EDTA anticoagulant tube and in 10 ml streck tube should be sent to Tongji laboratory; ^4^Blood samples should be obtained within 72 hours after two weeks of the first cycle of treatment; ^5^Blood samples should be obtained within 72 hours on Day 1, 15 and the last day of the second course of treatment; ^6^Tissue samples should be obtained intraoperatively and blood samples within 72 hours after operation; ^7^Blood samples should be obtained once before adjuvant chemotherapy after tumor reduction surgery; ^8^The third and sixth cycles of chemotherapy were obtained on Day 1; ^9^During the maintenance treatment of niraparib, follow-up was conducted every 12 weeks to obtain blood samples; ^10^During the long-term follow-up after the withdrawal of maintenance treatment, follow-up was conducted every 12 weeks, and evaluation was conducted every 24 weeks up to two years to obtain blood samples; ^11^Tissue samples should be obtained at the time of biopsy, and blood samples should be obtained within one week after confirming recurrence.

Recurrent tumor tissue and blood samples collected will be sent to Tongji central laboratory. A detailed sample collection process is presented in **Figure 3**. During the treatment period, patients will receive relevant examinations and are expected to complete questionnaires related to the QOL as per schedule. Post-treatment, patients will be subjected to follow-up examination and telephone follow-up. The detailed data obtained from the patients will be recorded in the CRF and kept strictly confidential in the research center. All study records and original documents will be maintained and stored according to relevant regulations and guidelines, or by the research institution’s rules. The investigator will access the relevant raw data of the clinical study and will be responsible for reviewing CRF to determine completeness, accuracy, and consistency of the information with the source data. Moreover, CRF, raw laboratory data, and medical test results must be readily available for clinical inspectors, auditors, and health authorities.

### Safety Analysis

The most common adverse events observed in a previous study were Anemia, Nausea, Thrombocytopenia, etc. and the serious (grade ≥3) adverse events were anemia, thrombocytopenia and neutropenia (19). All AEs will be monitored 30 days after the last dose in this study. The research coordinator or data manager will summarize all serious AEs resulting in treatment withdrawal or deaths during or within 30 days of treatment termination on a per-patient basis. AEs will be coded in accordance with Medical Dictionary for Drug Regulatory Activities (MedDRA). Patient’s survival and myelodysplastic syndrome/acute myeloid leukemia (MDS/AML) information will be collected every 8 weeks from the beginning of treatment up to 90 days after the end of the study. The safety set (SS) used for safety evaluation will include patients using niraparib at least once and with relatively complete medical records.

### Statistical Analysis

Hierarchical testing will be used to control the overall Type I error rate. First, ORR analysis will be conducted at the 1-sided alpha level of 0.05. If the result is positive, R0 resection rate analysis will be conducted with the 1-sided alpha level of 0.05. Descriptive measures will be used to summarize continuous variables (average value, standard deviation, median, maximum value, minimum value). Categorical variables will be expressed in frequency and percentage. The time to event analysis will be performed by the Kaplan Meier curve providing the median time to event. All data collected on CRF will be listed on a per-patient basis. Except for the date, the missing data will not be estimated. All statistical analysis will be calculated by SAS 9.4 statistical analysis software. Any deviations from the statistical methods given in the protocol will be reported in the final report as appropriate.

### Sample Size

Sample size will be determined based on an intended statistical power of 90% (one-sided test, significance level of 5%). ORR will be the first primary endpoint as it is more objective and better reflects the effectiveness of niraparib neoadjuvant therapy. Based on the assumption that niraparib will be considered ineffective if the ORR is ≤ 20% (P0), and effective if the ORR is ≥ 40% (P1), this study can further proceed to large-scale clinical trials. The proposed study will utilize Simon’s two-stage design wherein in stage 1 a total of 24 patients will be enrolled. If the number of patients achieving objective response is >5, then the study will proceed to stage 2. In stage 2, a total of 21 patients were planned to be enrolled. A total of 53 patients will be included in the study, considering a possible 15% drop-out rate of patients. The intended ORR for achieving the primary endpoint in stage 2 will be >8 patients with objective response. Once the study achieves the first primary objective, the R0 resection rate of the second primary endpoint will be analyzed sequentially. Using 45 sample size, one-sided test, significance level of 5%, power was calculated to be 85.7%. Finally, the actual R0 resection rate and the confidence interval will be obtained according to statistical analysis of the data.

## Discussion

To the best of our knowledge, this will be the first prospective multicenter study to evaluate the safety and effectiveness of niraparib alone as neoadjuvant treatment in advanced OC. The results from this study may propose a new treatment alternative for HRD positive patients with OC and extend the therapeutic applications of PARPi. Currently, not much is known about response of treatment-naïve patients to PARPi, and this long-lasting unsolved question has troubled many researchers.

So far, platinum-based chemotherapy is the only acceptable option for neoadjuvant treatment in patients with advanced OC (20). This study may extend the neoadjuvant treatment strategies in OC. Notably, the use of NACT for patients with OC increased from 17.6% in 2004 to 45.1% in 2016 (21). However, platinum- and paclitaxel-based NACT failed to grant any survival benefits in all existing randomized controlled trials (RCTs), leading to serious concerns on the effectiveness of NACT (10, 22). Oncogene targeted therapies have been proved to be more efficacious and safer than chemotherapy in serval other types of tumors, such as lung and breast cancer leading to a significantly improved prognosis and quality of life (23, 24). Similar therapies are rarely available for OC. Previously, ANTHALYA and GEICO 1205/NOVA clinical trials provided evidence on bevacizumab as a NACT in addition to chemotherapy in advanced OC, suggesting limited improvement in ORR (25). Most recently, the NUVOLA trial is recruiting patients to evaluate neoadjuvant olaparib and weekly TC (carboplatin plus paclitaxel) in unresectable OC (26). Therefore, it is imperative to explore more possibilities for neoadjuvant treatment in OC. Keeping in view the facts discussed above, our study was designed to find out whether niraparib neoadjuvant treatment could reduce surgical complexity and improve patients’ prognosis in advanced OC. Besides, this study could provide a foundation for future RCTs aiming to evaluate the potential PFS improvement with neoadjuvant niraparib therapy in patients with HRD positive OC.

The primary endpoint chosen for this study will ensure the maximum clinical translational potential since it represents the neoadjuvant treatment response as well as the complexity of the debulking surgery. Meanwhile, ORR, PFS, and OS could depict the anti-tumor potential of niraparib comprehensively in the short- and long-term respectively. Additionally, the sample size was calculated using Simon’s two-stage design. If niraparib was proven effective in a small population in the first stage, then only this study can proceed to the second stage and recruit more patients. More importantly, considering the safety concerns for the enrolled patients, serum CA125 levels will be measured biweekly. This will partially reflect the treatment responses and ensure patients with uncontrolled diseases withdrew from the experimental treatment on time. This design will not only secure the greatest clinical benefit to participants but also establish a scientifically reliable trial.

Several limitations of the study design can be acknowledged. Firstly, as of now, only Chinese patients will be included in the study. Secondly, no control group exists in this study, which should be notified during the result explanation and further appended in future studies.

Generally, this study may assess the potential of niraparib neoadjuvant treatment and IDS as a valid therapeutic strategy for patients with unresectable bulky tumors or poor general conditions. International investigators are welcome to contact and collaborate so that patients other than of Chinese origin can be enrolled in the study.

## Data Availability Statement

The original contributions presented in the study are included in the article/supplementary material. Further inquiries can be directed to the corresponding authors.

## Author Contributions

QG, DZ, and JL developed the study concept and protocol. HL and RL assisted in further development of the protocol. YH, LH, DM, and QG are responsible for the supervision of the clinical trial. QG has access to the final trial dataset. All authors contributed to the article and approved the submitted version.

## Funding

This trial is funded by Pioneer Research Foundation of Tongji Hospital.

## Conflict of Interest

The authors declare that the research was conducted in the absence of any commercial or financial relationships that could be construed as a potential conflict of interest.

## Publisher’s Note

All claims expressed in this article are solely those of the authors and do not necessarily represent those of their affiliated organizations, or those of the publisher, the editors and the reviewers. Any product that may be evaluated in this article, or claim that may be made by its manufacturer, is not guaranteed or endorsed by the publisher.

## Abbreviations

OC, Ovarian cancer; FIGO, The International Federation of Gynecology and Obstetrics; NACT, Neoadjuvant chemotherapy; IDS, Interval debulking surgery; Poly (ADP-ribose) polymerase (PARP) inhibitors; FDA, Food and Drug Administration; HRD, Homologous recombination deficiency; ORR, Objective response rate; PFS, Progression-free survival; CR, Complete response; PR, Partial response; SD; Stable disease; AEs, Adverse events; OS, Overall survival; GCP, Good Clinical Practices; CT, Computed tomography; RECIST, Response Evaluation Criteria in Solid Tumors; CTCAE, Common Terminology Criteria for Adverse Events; DCR, Disease control rate; CA125, Cancer antigen 125; GCIC, Gynecologic Cancer Intergroup Consensus; CFI, Chemotherapy-free interval; TFST, Time to first subsequent therapy; QOL, Quality of life; PRO, Patient report outcome; FACT-O, Functional Assessment of Cancer Therapy-Ovarian; HADS, Hospital Anxiety and Depression Scale; ISI, Insomnia Severity Index; IPAQ, International Physical Activity Questionnaire; EQ-VAS, EuroQol-visual analog scales; LOH, Loss of heterozygosity; TAI, Telomeric allelic imbalance; LST, Large scale state transitions; CRF, Case report form.
